# The Six Year Molars

**Published:** 1887-11

**Authors:** A. J. Smith


ARTICLE IV.

### THE SIX YEAR MOLARS.

BY A. J. SMITH, D. D. S.

*Mr. President, Ladies and Gentlemen*: There are, perhaps, no teeth about which more has been said than the first permanent molars; and there are none more in need of further consideration, because there are none more valuable and none more neglected. My subject, therefore, "though old and threadbare," is yet one of importance.

I will not presume to advise what is best to be done for these teeth in their different conditions, but will briefly tell you what I do for those that come under my care. There is no operation in the practice of dentistry that so appeals to my sympathies, or goes so against my inclinations, as the extraction of these teeth for children.

First permanent molars coming under my care soon after the second ones have erupted, I fill, if they give promise of lasting three or four years, telling the patient that if we can save them only that long we may then know of some better way of saving them longer, and will prevent the second permanent molar from tipping forward. That will be worth more than the cost of filling the other tooth. Yet, like all other rules for these teeth, there are exceptions to this one. If the pulp is dead and periosteum and gum inflamed, I generally extract at once; or if the teeth are already crowded and some irregularity exists that additional room will correct, I consider that extraction is indicated.

The treatment of the first permanent molars, after the second ones have erupted, is an easy matter compared with the treatment of them before. These teeth are so imperfect and incomplete in structure as to render them susceptible to caries, and they appear at an age when the child has barely emerged from infancy, and still requires the gentle handling demanded by tender years.

It frequently happens that the child's first introduction to the dentist is brought about by the aching of one of these teeth. Only a few days ago a father came to me with his little delicate seven-year-old son to have something done for the toothache. Upon examination I found the trouble to be in the left inferior first permanent molar, which was in a deplorable condition; the enamel entirely gone, except a small ledge on the buccal surface; the dentine a pulpy mass of about the consistency of cork; pulp nearly exposed and highly inflamed; had been aching for three days. This was the child's first acquaintance with a dentist, but he had a very distinct recollection of meeting a physician only a few days previous, who mistook the mate to this one, the right inferior first permanent molar, for a temporary tooth, and attempting to extract it as such, broke it off just below the gum, leaving a live pulp exposed, and the boy with a horror of having a tooth extracted. I advised the parent that this tooth was beyond redemption, and the only means of relief in extraction; upon which the boy's courage seemed to fail at once, and he began crying and vowed he would not have it out. I suggested giving the boy an anæsthetic and taking the tooth out without pain, as it would be a pretty severe operation, and in the face of the misfortune he had experienced with the other one, I did not wonder at his not having the courage to undertake this operation.

But the father opposing the anæsthetic, I told the little fellow I would put something in it to keep it from hurting for a while, and see if we could possibly save it, my object being to gain the boy's confidence and get him and the tooth in better condition for the extraction. After removing as much of the pulpy dentine as I could without much pain, and drying the remainder with cotton chloroform and the chip blower, I saturated it with carbolic acid and placed a rubber ligature around the neck of the tooth and dismissed the little patient until it gave him further trouble. The next day they returned, with the opinion that the tooth could not be saved, and as it was considerably loosened, with a little encouragement the boy consented to have it out. The operation was more severe than the child expected yet it was much easier than it would have been but for the work of the ligature.

First permanent molars, giving trouble by the 7th or 8th year, I, as a rule, endeavor to save until the 9th or 10th year, if I can get them before the pulp dies or has to be destroyed, as frequently the deficiency of ossific material will have been supplied by this time and the tooth then may be permanently filled; and, if not, a child is better able to stand it and can better afford to lose it then than sooner; and it will yet be in time to allow the second molar to come forward bodily, instead of tipping.

Yet, there are cases, like the one described, in which there is no opportunity to give even temporary relief, and extraction seems the only resource; in such cases I consider it the part of kindness and humanity to use an anæsthetic, as children possess thorough immunity from the dangers of them. Besides, it is desirable to avoid severe pain with children that we may not create a life-long dread of dental operations, which is so often due to the keen remembrance of suffering in childhood.

I have no definite or specific rule for extracting or saving first permanent molars, nor do I think any is practicable. What seems to me to be far more needed is to instruct and educate the parents to have their children's teeth cared for in time. Many parents think no attention is necessary to the temporary teeth, and know nothing at all about the first permanent molars until they are badly decayed and aching, and are informed by the dentist that they are permanent teeth. Then they say, "I thought they were baby teeth, or I would have had them filled long ago." Hence, the importance of parents knowing the necessity of having their children's teeth cared for at an early age.

The almost total lack of popular dental literature giving instruction on the most important of all subjects that effect the health and comfort of the people, is not altogether creditable to the dental profession, for it is to the dentists that people must look for right teaching along this line.

About the only hope of restoring perfect teeth to future generations, lies in the care and intelligent use of means placed in the reach of the mothers. To them, and not to the dentist, is committed the trust and responsibility of remedying many of the defects from which we now suffer, but the duty of proper instructions is with the dentist. Now, some one may say that for us to give this instruction is an endless job for which we would get poor pay. So it may seem to many of you at first thought, but if each of us would go to work earnestly, and embrace each opportunity of imparting a few words of advice to parents regarding early attention to their children's teeth, in a very few years we would be able to see progress, not only by better teeth of children, but by a higher appreciation of our profession by the public generally.

One writer on this subject has said that the time will come when we as dentists shall feel largely responsible for the original character of children's teeth, that we are mere workmen in the art of dentistry, and so, subordinately professors in its science; that we feel little responsibility for instructing our patients. We call our patrons patients, but they come to us more as customers, and therefore only for our skill. But when they come to us for advice and we are able to sustain ourselves as teachers, we will have a higher claim to the title of professional gentlemen.

Our relation to those we serve and to the public should be of so much confidence, dignity and learning, that mothers will come to us for instruction to prevent bad teeth in their children, that shall reach their own habits prior to their children coming into the world. Our patients should be made to feel that our advice is worth as much as our skill. But this can be established only subsequently to the education of the people.—*Transactions of the Indiana State Dental Association.*

